# Model Updating of Complex Structures Using the Combination of Component Mode Synthesis and Kriging Predictor

**DOI:** 10.1155/2014/476219

**Published:** 2014-01-22

**Authors:** Yang Liu, Yan Li, Dejun Wang, Shaoyi Zhang

**Affiliations:** ^1^School of Transportation Science and Engineering, Harbin Institute of Technology, Harbin 150090, China; ^2^Postdoctoral Station of Civil Engineering, Harbin Institute of Technology, Harbin 150090, China

## Abstract

Updating the structural model of complex structures is time-consuming due to the large size of the finite element model (FEM). Using conventional methods for these cases is computationally expensive or even impossible. A two-level method, which combined the Kriging predictor and the component mode synthesis (CMS) technique, was proposed to ensure the successful implementing of FEM updating of large-scale structures. In the first level, the CMS was applied to build a reasonable condensed FEM of complex structures. In the second level, the Kriging predictor that was deemed as a surrogate FEM in structural dynamics was generated based on the condensed FEM. Some key issues of the application of the metamodel (surrogate FEM) to FEM updating were also discussed. Finally, the effectiveness of the proposed method was demonstrated by updating the FEM of a real arch bridge with the measured modal parameters.

## 1. Introduction

FEM updating of structures is to correct the modeling errors of the analytical FEM by using the measured data, and this technique is applied to generate a refined baseline FEM that accurately predicts the dynamic or static behavior of structures. For this reason, FEM updating has a wide application to many research fields related to civil structures such as damage detection, vibration control, and dynamic response analysis. FEM updating of structures usually ends up with a nonlinear optimization problem, and many methods have been developed to address the issue of FEM updating [1, 2]. Needless to say, amounts of iterative computation are inevitable for updating the FEM of complex structures such as the practical long-span bridges, because the complicated structures usually lead to more serious nonlinear optimization issue of FEM updating. For the large-scale structures, however, it is time-consuming even just for one-step dynamic analysis. The regular methods are difficult or even impossible to implement. Therefore, it is necessary and essential to figure out a cheaper alternative to costly FEM analysis in order to effectively update the FEM of the large-scale and complex structures.

The difficulty and the low efficiency of FEM updating of complex structures mainly lie in the large number of degrees of freedom (DOF) and the huge size of FEM. Therefore, it is effective to improve the efficiency of FEM updating by the “indirect usage” of the analytical FEM. Herein, the “indirect usage” means to generate a surrogate model (metamodel) of the analytical FEM of structures, which is the relationship between the structural characteristics and the structural parameters. The metamodel usually takes a mathematical function with some coefficients obtained by regression method to be the approximation of the above functional relationship. With this idea, the dynamic analysis of the analytical FEM could be replaced by this metamodel. Different applications in many fields go to three main types of metamodel, that is, artificial neural network (ANN) [3], Kriging method [4], and response surface method (RSM) [5]. Among these three kinds of metamodel, in this paper, the Kriging predictor is applied to FEM updating, and the reason for selecting this metamodel is described in Section 4.

Although the metamodel is an effective alternative to the analytical FEM of structures, it still needs the analytical FEM to do dynamic analysis repeatedly for couple of times in order to build the metamodel. However, for the large-scale structures, it is difficult to carry out the dynamic analysis such as the structural eigendecomposition even just for one time. Therefore, it is necessary to reduce the size of the analytical FEM reasonably before the generation of metamodel, and CMS is a good option for this purpose. CMS technique is quite effective in executing the dynamic analysis of large-scale structures, and this method not only reduces the scale of structural eigenanalysis but also ensures the accuracy of solution [6–8]. One thing needed to mention is that the CMS method, to some extent, also could be applied to solve the issue of large-scale FEM updating. However, the metamodel is more efficient than CMS method [9] since the size of condensed FEM obtained by CMS method cannot be infinite small in order to ensure the accuracy of condensed FEM. Therefore, we recommend to obtain a reasonable condensed FEM of complex structures by using the CMS technique firstly, and then the metamodel is generated based on this condensed FEM. The generated metamodel is apt to deal with amounts of iterative calculation during the optimization search of FEM updating.

Based on the discussion above, it is deserved to combine the metamodel with CMS technique for the FEM updating of complex structures, which was not so much investigated. Therefore, a two-level method for FEM updating of large-scale and complex structures is proposed in this study. In the first level, the structural eigendecomposition of the reduced FEM is carried out by the CMS method. The Kriging model is applied to handle the large number of iterative searches for FEM updating in the second level.

To this end, the remainder of this paper is organized as follows. The basic idea of FEM updating is introduced briefly in the next section. The detail of the CMS method is described in Section 3. Kriging model is discussed, and some key issues of the application of metamodel to the FEM updating are investigated in Section 4. And then the effectiveness of the proposed method is identified by updating the FEM of a practical arch bridge. At last, conclusions are drawn.

## 2. Finite Element Model Updating

FE model updating of structures aims to correct the modeling error of the analytical FE model by minimizing the difference between the measured static or dynamic data and the results obtained by the analytical FE model. The difference between the measured and analytical results is defined as the following equation:

(1) $$\begin{array}{c} J=\varepsilon^{T}W_{\varepsilon }\varepsilon , \end{array}$$

where **W**_*ε*_ is the weighing matrix, and the usual form is defined as

(2) $$\begin{array}{c} W_{\varepsilon }={\left[\mathrm{diag}\left({Ζ}_{m}\right)\right]}^{-2}, \end{array}$$

where **Ζ**_*m*_ represents the measured results such as the modal parameters and [diag⁡(·)] and diag⁡(·) represent the diagonal matrix and the principal diagonal elements of matrix, respectively. The **ε** in (1) means the difference between the measured results and the analytical results, which is defined as

(3) $$\begin{array}{c} \varepsilon ={Ζ}_{m}-Ζ\left(\theta \right), \end{array}$$

where **Ζ**(***θ***) is the analytical static or dynamic characteristics of structures and ***θ*** are the updating parameters for FE model updating. With the first-order Taylor series, the approximation to (3) is obtained by

(4) $$\begin{array}{c} Ζ\left(\theta \right)\approx Ζ\left(\theta_{i}\right)+G\left(\theta_{i}\right)\left(\theta -\theta_{i}\right), \end{array}$$

where ***θ***_*i*_ is the updating parameter at the *i*th iteration and **G**(***θ***_*i*_) is the derivative of **Ζ**(***θ***) with respect to ***θ***_*i*_.

The optimal estimate of the updating parameters could be obtained by solving the optimization problem shown in (1) by using the least square method. The unique optimal solution of (1) depends on the full column rank of the sensitivity matrix **G**, and this condition always cannot be satisfied, which means the number of updating parameters is bigger than the number of measured structural characteristics or some selected updating parameters are correlated. Therefore, the regularization methods [10] are always applied to add some additional constrains to (1) such as the following equation:

(5) $$\begin{array}{c} J=\varepsilon^{T}W_{\varepsilon }\varepsilon +\gamma^{2}{\left(\theta -\theta_{0}\right)}^{T}W_{\theta }\left(\theta -\theta_{0}\right), \end{array}$$

where *γ* represents the regularization factor, and the weighting matrix **W**_*θ*_ is defined as [11]

(6) Wθ=mean(diag⁡(G(θi)TWεG(θi)))mean(diag⁡([G(θi)TWεG(θi)]−1))·[diag⁡(G(θi)TWεG(θi))]−1.

There are couples of methods to determine the regularization factor *γ*, and a common method is the L-curve method. From the multiobjective optimization point of view, the above issue could be defined as a two-objective optimization function, that is,

(7) $$\begin{array}{c} \min\left\{J_{1},J_{2}\right\},\;J_{1}=\varepsilon^{T}W_{\varepsilon }\varepsilon ,\;\;J_{2}={\left(\theta -\theta_{0}\right)}^{T}W_{\theta }\left(\theta -\theta_{0}\right). \end{array}$$

The optimal problem defined in the above equation has similar meaning to the objective function shown in (5) since the generation of a reasonable value *γ* is closely related to find a compromise estimate of ***θ*** satisfying the two-objective function shown in (7).

In this study, we investigate building a surrogate model of the analytical FE model, so the characteristics of structures **Ζ**(***θ***) shown in (3) are obtained by this surrogate model (metamodel) instead of the FE model analysis. With this procedure, the efficiency of FE model updating could be improved dramatically.

## 3. Component Mode Synthesis Method 

The basic idea of CMS method is that the dynamic characteristics of the whole structure could be obtained by the combination of the results acquired from different substructure. Comparing with the dynamic analysis of the whole structure, the dynamic analysis of each substructure is easy to be implemented. Therefore, the eigendecomposition of complex structures could be carried out efficiently by using the CMS method. The procedure of CMS method is described as follows:

- divide the whole structure into different substructures or components;
- implement the dynamic analysis of each component;
- build the condensed FEM depending on the boundary conditions applied to the substructure interfaces;
- obtain the structural frequencies and modal shapes of the condensed FEM;
- transform the modal shapes at the condensed coordinate to the original physical coordinate.

**Figure 1:**
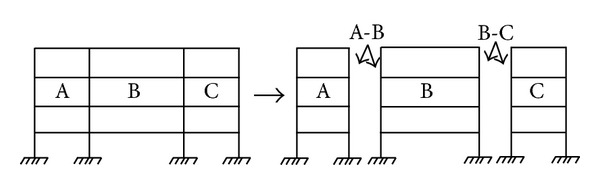
The whole structure and three substructures.

In this paper, an improved CMS method [12] is utilized to get the reduced FEM of complex structures. For convenience, a frame structure shown in Figure 1 is taken as an example to describe the procedure of this CMS method.

Ignoring the effect of damping, the vibration equation of the frame structure can be defined by

(8) $$\begin{array}{c} M\ddot{x}+Kx=f, \end{array}$$

where **M** and **K** represent the mass matrix and stiffness matrix of the frame structure, respectively, and **f** and **x** describe the force vector and displacement vector, respectively.

As shown in Figure 1, three substructures are designated as A, B, and C. Furthermore, let the DOF of non-interface of A, B, and C be represented by the suffixes 1, 3, and 5 respectively. In addition, let the DOF of the interface among A-B and B-C be expressed by 2 and 4, respectively. With these suffixes, the matrixes **M** and **K** in (1) are written as follows:

(9) $$\begin{array}{c} K=\left[\begin{array}{ccccc} K_{11} & K_{12} & 0 & 0 & 0\\ K_{21} & K_{22} & K_{23} & 0 & 0\\ 0 & K_{32} & K_{33} & K_{34} & 0\\ 0 & 0 & K_{43} & K_{44} & K_{45}\\ 0 & 0 & 0 & K_{54} & K_{55} \end{array}\right],\\ M=\left[\begin{array}{ccccc} M_{11} & M_{12} & 0 & 0 & 0\\ M_{21} & M_{22} & M_{23} & 0 & 0\\ 0 & M_{32} & M_{33} & M_{34} & 0\\ 0 & 0 & M_{43} & M_{44} & M_{45}\\ 0 & 0 & 0 & M_{54} & M_{55} \end{array}\right]. \end{array}$$

In the CMS method, we assume that the modal shapes of the whole structure are the superposition of the modal shapes of all the substructures and the displacement of the interface. The vibration modal shape of each substructure is obtained by solving the following eigenequation:

(10) $$\begin{array}{c} K_{ii}\Phi_{i}=\Lambda_{i}M_{ii}\Phi_{i},\;\left(i=1,3,5\right), \end{array}$$

where Λ_*i*_ and Φ_*i*_ represent the eigenvalue and eigenvector of *i*th component, respectively.

Depending on the two components adjacent to the same interface, the modal shapes of the interface are obtained by the following equation:

(11) [K11K120K21K22K230K32K33][Φ1Φ2Φ3] =[Λ1000Λ2000Λ3][M11M120M21M22M230M32M33][Φ1Φ2Φ3],

where Φ_*i*_ is the *n* modes starting from low order, that is,

(12) $$\begin{array}{c} \Phi_{i}=\left[\begin{array}{cccc} \varphi_{i1} & \varphi_{i2} & \cdots & \varphi_{in} \end{array}\right],\;\left(i=1,3,5\right). \end{array}$$

According to (11) and (12), the *n* modes of interface starting from low order are obtained such as the modes of A-B interface:

(13) $$\begin{array}{c} \left[\begin{array}{c} \Phi_{1}^{\text{AB}}\\ \Phi_{2}^{\text{AB}}\\ \Phi_{3}^{\text{AB}} \end{array}\right]=\left[\begin{array}{cccc} \Phi_{11}^{\text{AB}} & \Phi_{12}^{\text{AB}} & \cdots & \Phi_{1n}^{\text{AB}}\\ \Phi_{21}^{\text{AB}} & \Phi_{22}^{\text{AB}} & \cdots & \Phi_{2n}^{\text{AB}}\\ \Phi_{31}^{\text{AB}} & \Phi_{32}^{\text{AB}} & \cdots & \Phi_{3n}^{\text{AB}} \end{array}\right]. \end{array}$$

Accordingly, the condensed structure can be obtained by the *n* eigenmode of whole structure starting from low order, which is described in the following equation.

(14) $$\begin{array}{c} T^{T}KT\eta =\Lambda T^{T}MT\eta , \end{array}$$

where **η** represents the displacement of condensed FEM and **T** is the matrix transforming the condensed coordinate to the original physical coordinate, that is,

(15) $$\begin{array}{c} \left[\begin{array}{c} x_{1}\\ x_{2}\\ x_{3}\\ x_{4}\\ x_{5} \end{array}\right]=T\left[\begin{array}{c} \eta_{1}\\ \eta_{2}\\ \eta_{3}\\ \eta_{4}\\ \eta_{5} \end{array}\right]=T\eta , \end{array}$$

where **T** is defined as

(16) $$\begin{array}{c} T=\left[\begin{array}{ccccc} \Phi_{1} & 0 & 0 & \Phi_{1}^{\text{AB}} & 0\\ 0 & 0 & 0 & \Phi_{2}^{\text{AB}} & 0\\ 0 & \Phi_{3} & 0 & \Phi_{3}^{\text{AB}} & \Phi_{3}^{\text{BC}}\\ 0 & 0 & 0 & 0 & \Phi_{4}^{\text{BC}}\\ 0 & 0 & \Phi_{5} & 0 & \Phi_{5}^{\text{BC}} \end{array}\right]. \end{array}$$

Therefore, the modal frequencies of the whole structure can be obtained by (14), and the modal shapes can be acquired by (15).

## 4. Kriging Model

The basic idea of metamodel is to build a surrogate model by using regression technique with limited feature samples, and different mathematical form goes to different metamodel. The procedure of building the metamodel consists of three parts, that is,

- selection of the feature samples in structural parameters space;
- fitting the mathematical functional model by using the selected feature samples;
- assessment of the generated metamodel. The generation of Kriging model also needs these three procedures described in detail as follows.

### 4.1. Selection of Feature Samples

Selection of the feature samples is also called design of experiments (DOE). The response function (the dynamic characteristics of structures) is obtained by substituting the selected samples of structural parameters into the analytical FE model, and then the response function and the samples of structural parameters are applied to build the metamodel. Therefore, the proper selected samples are essential for the efficiency and the accuracy of generated metamodel. Couples of methods have been applied to select the feature samples such as full factorial design [13], fractional factorial design [14], central composite design [4], and orthogonal array [15].

Many previous works have been carried out in order to investigate the relative merits of different DOE, but the opinions on the appropriate selection of DOE always varied case by case. For the deterministic computer analysis such as FEM analysis, the only consensus reached so far is that the feature samples should be “spaced filling” which means treating the whole sample space equally as far as possible. Based on this idea, some methods were developed to generate the feature samples, and among them, the Latin hypercube sampling [16] is a popular strategy for generating random sample points ensuring that all portions of the sample space is represented. Herein, this method are described briefly as follows.

Assume the set of feature samples of updating parameters is Γ = [***θ***_1_,***θ***_2_,…,***θ***_*N*_]^*T*^ and each vector of updating parameters is ***θ***_*v*_ = {*θ*_*v*1_,*θ*_*v*2_,…,*θ*_*vq*_}^*T*^(*v* = 1,2,…, *N*) (*N* is the number of total samples, and *q* is the number of updating parameters). The corresponding dynamic characteristics of structures obtained by the analytical FEM are **Ζ**_*j*_(Γ) = {*Z*_*j*_(***θ***_1_),*Z*_*j*_(***θ***_2_),…,*Z*_*j*_(***θ***_*N*_)}^*T*^  ( *j* = 1,2, …, *p*, and *j* represents the number of measured modal parameters). The procedure of Latin hypercube consists of three parts; that is,

- divide the interval of ***θ***_*v*_ into *r* nonoverlapping intervals having equal probability such as the uniform distribution;
- sample randomly from a uniform distribution a point in each interval at each dimension;
- pair randomly (equal likely combinations) the point from each dimension.

### 4.2. Mathematical Form of the Kriging Model

Assume the whole set of samples is **S** and the set of selected feature samples is the subset of **S**, that is, Γ = ([***θ***_1_,***θ***_2_,…,***θ***_*N*_]^*T*^)_*N*×*q*_, and each element of Γ is defined as ***θ***_*i*_ ∈ *R* ^*q*^  (*i* = 1,2,…, *N*). The response function is **Z** = ([**Ζ**_1_,…,**Ζ**_*N*_]^*T*^)_*N*×*p*_, and **Ζ**_*j*_(Γ) = {*Z*_*j*_(***θ***_1_),*Z*_*j*_(***θ***_2_),…,*Z*_*j*_(***θ***_*N*_)}^*T*^(*j* = 1,2,…, *p*). With the normalization method, the mean and variance of the selected feature samples are defined as

(17) $$\begin{array}{c} E\left(\theta_{k}\right)=0,\;\mathrm{Var}\left(\theta_{k}\right)=1,\;\left(k=1,2,\ldots ,q\right),\\ E\left({Ζ}_{j}\right)=0,\;\mathrm{Var}\left({Ζ}_{j}\right)=1,\;\left(j=1,2,\ldots ,p\right), \end{array}$$

where *E*(·) and *V*(·) represent the mean and variance, respectively.

With the Kriging model, the estimation of dynamic characteristic of structures *Z*_*j*_(***θ***_*k*_) is divided into two parts, that is, the trend term and the random term, which is defined as [17, 18]

(18) $$\begin{array}{c} {\hat{Z}}_{j}\left(\theta_{v}\right)=f_{j}{\left(\theta_{v}\right)}^{T}\beta_{j}+\gamma_{j}\left(\theta_{v}\right),\;\left(v=1,2,\ldots ,N\right), \end{array}$$

where **f**_*j*_(***θ***_*v*_) is usually a polynomial and **β**_*j*_ is the coefficient vector. Since ***θ***_*v*_ is usually multivariable, the random item *γ*_*j*_(***θ***_*v*_) is a random process whose mean and covariance are described as follows. The mean is defined as

(19) $$\begin{array}{c} E\left(\gamma_{j}\left(\theta_{v}\right)\right)=0. \end{array}$$

And the covariance is defined as,

(20) E(γj(θv)·γj(w))=Var⁡(γj(θv))·Cor(ρj,θd,θv), (d=1,2,…,N),

where **w** ∈ **S** and **w** ∈ Γ and Cor(·) is the correlation function with respect to **ρ**_*j*_ = {*ρ*_*j*1_,*ρ*_*j*2_,…,*ρ*_*jq*_}^*T*^. If **w** ∉ Γ, the above covariance is defined as

(21) $$\begin{array}{c} E\left(\gamma_{j}\left(\theta_{v}\right)\cdot \gamma_{j}\left(w\right)\right)=\mathrm{Var}\left(\gamma_{j}\left(\theta_{v}\right)\right)\cdot \text{Cor}\left(\rho_{j},w,\theta_{v}\right). \end{array}$$

The correlation function shown in the above two equations has no unique form, and the common correlation function is the Gaussian function, that is,

(22) $$\begin{array}{c} \text{Cor}\left(\theta_{d},\theta_{v}\right)=\exp\left\{-\sum_{k=1}^{q}\rho_{k}{\left|\theta_{dk}-\theta_{vk}\right|}^{2}\right\}. \end{array}$$

With above equations, the coefficients of the Kriging model could be estimated by optimization method, and the detail of the estimation of these coefficients is described in the appendix.

### 4.3. Assessment of the Kriging Model

To assess the accuracy of the generated Kriging model, a common way is to compare the error between the true values and estimated response function at extra test samples. The index SST (total sum of squares) [19] is defined as

(23) $$\begin{array}{c} \text{SST}=\sum_{u=1}^{K}{\left(Z_{j}\left(\theta_{u}\right)-\overset{-}{Z}\left(\Gamma_{a}\right)\right)}^{2}, \end{array}$$

where $\overset{-}{Z}\left(\Gamma_{a}\right)=\left(Z_{j}\left(\theta_{1}\right)+Z_{j}\left(\theta_{2}\right)+\cdots +Z_{j}\left(\theta_{K}\right)\right)/K$, Γ_*a*_ = {***θ***_1_,***θ***_2_,…,***θ***_*K*_}^*T*^, and *K* is the number of extra test samples. The above equation is simplified by

(24) $$\begin{array}{c} \text{SST}=Ζ{\left(\Gamma_{a}\right)}^{T}Ζ\left(\Gamma_{a}\right)-\overset{-}{Z}{\left(\Gamma_{a}\right)}^{2}. \end{array}$$

The index SST is composed of two parts, that is, SSR (sum square of regression) and SSE (sum square of error).

(25) $$\begin{array}{c} \text{SST}=\text{SSR}+\text{SSE}. \end{array}$$

SSR is defined as

(26) $$\begin{array}{c} \text{SSR}=\sum_{u=1}^{K}{\left({\hat{Z}}_{j}\left(\theta_{u}\right)-\overset{-}{Z}\left(\Gamma_{a}\right)\right)}^{2}=b\Theta^{T}Ζ\left(\Gamma_{a}\right)-\overset{-}{Z}{\left(\Gamma_{a}\right)}^{2}. \end{array}$$

SSE is defined as

(27) SSE=∑u=1K(Zj(θu)−Z−(Γa))2=(Ζ(Γa))TΖ(Γa)−bΘTΖ(Γa).

With the SSR and SSE, the following index is defined as

(28) $$\begin{array}{c} \text{R}{\text{S}}^{2}=1-\frac{\text{SSE}}{\text{SSR}},\;\left(0\leq \text{R}{\text{S}}^{2}\leq 1\right). \end{array}$$

RS^2^ is applied to evaluate the confidence extent of the generated metamodel. The closer to 1 the value is, the high confidence the metamodel has. Usually, the above procedure needs many extra test samples which is difficult to be realized in practice. Therefore, the leave-*h*-out [20] cross evaluate strategy is a good choice to reduce the number of test samples. The procedure of the leave-*h*-out cross-validation is a loop process that is described as follows.

- Take *h* samples from the set of training samples and treat these *h* samples as the test samples.
- Build the metamodel with the rest of training samples.
- Evaluate the confidence level of the generated metamodel by using the test samples.
- Repeat the above three steps until all the different *h* samples extracted from the set of training samples are deemed as test samples.
- Take the mean of all the generated metamodel as the final metamodel.

### 4.4. Determining of the Mathematical Form of Kriging Model

With the variance analysis, the mathematical form of the RSM could be determined. Taking (18) as an example, if we assume that both SSR and SSE satisfy the Gauss distribution, the following index [21] is defined with the *F*-test:

(29) $$\begin{array}{c} {\text{Fs}}_{k}=\frac{\text{SS}{\text{R}}_{k}/q}{\text{SS}{\text{E}}_{k}/\left(N-q-1\right)},\;\left(k=1,2,\ldots ,q\right), \end{array}$$

where *q* and *N* − *q* − 1 represent the DOF of SSR_*k*_ and SSE_*k*_, respectively.

Since Fs satisfies the *F*-distribution, if the value of Fs_*k*_ is bigger than *F*_*α*__,*q*,*N*−*q*−1_ under the given confidential level *α*, this term has significant contribution to the response function. This term will be kept in the mathematical function or will be deleted for its little contribution to the response function. Repeating this procedure, the mathematical form of the Kriging model could be determined. The procedure of the generation of Kriging model is described as follows:

- select the feature set of samples Γ by using the feature selection method;
- with the FE model analysis, obtain the response variable **Ζ**(Γ) with respect to selected feature samples;
- assume the initial polynomial and correlated function of Kriging model;
- obtain the coefficient vector **ρ** of Kriging model by solving the optimization problem defined in the appendix (see ((A.15)), and then determine β* and Var⁡(**γ**(***θ***_*v*_)) shown in ((A.14));
- determine the test set of samples, and calculate the SSR and SSE by using (26) and (27);
- determine the final mathematical form of Kriging model by using Fs obtained from (29);
- assess the accuracy of the generated Kriging model by using the method in Section 4.3; if the accuracy is good enough, output the results or go back to step (i).

### 4.5. Some Key Issues of the Application of Metamodel to FEM Updating 

#### 4.5.1. Statistical Meaning of the Generated Metamodel

Another popular metamodel is RSM. A key issue that is easy to be ignored is proposed by comparing the performance of Kriging model and RSM. The RSM always takes the polynomial form, that is,

(30) $$\begin{array}{c} {Ζ}_{j}\left(\Gamma \right)=\Theta \beta +\varepsilon_{L}, \end{array}$$

where **ε**_*L*_ is the error term and

(31) $$\begin{array}{c} {Ζ}_{j}\left(\Gamma \right)=\left[\begin{array}{c} Z_{j}\left(\theta_{1}\right)\\ \vdots \\ Z_{j}\left(\theta_{N}\right) \end{array}\right],\;\;\Theta =\left[\begin{array}{cccc} 1 & \theta_{11} & \theta_{12} & \cdots \\ 1 & \theta_{21} & \theta_{22} & \cdots \\ \vdots & \vdots & \cdots & \cdots \\ 1 & \theta_{N1} & \theta_{N2} & \cdots \end{array}\right],\\ \beta =\left[\begin{array}{c} \beta_{0}\\ \beta_{1}\\ \vdots \end{array}\right],\;\;\varepsilon_{L}=\left[\begin{array}{c} \varepsilon_{L1}\\ \vdots \\ \varepsilon_{LN} \end{array}\right]. \end{array}$$

Utilizing the generated RSM, the estimated set of characteristics of structures is obtained as

(32) $$\begin{array}{c} {\hat{Ζ}}_{j}\left(\Gamma \right)=\Theta b+\varepsilon_{L}, \end{array}$$

where vector **b** is the estimated vector of the vector **β**, and this is obtained by the least squared method, that is,

(33) $$\begin{array}{c} b={\left(\Theta^{T}\Theta \right)}^{-1}\Theta^{T}{\hat{Ζ}}_{j}\left(\Gamma \right). \end{array}$$

Since the RSM is based on the statistical theory, the error term in (30) consists of two parts, that is, modeling error and random error, respectively,

(34) $$\begin{array}{c} {\hat{Ζ}}_{j}\left(\Gamma \right)=\Theta b+\varepsilon_{L1}+\varepsilon_{L2}, \end{array}$$

where **ε**_*L*1_ represents the modeling error caused by the difference between the RSM and the true model and **ε**_*L*2_ represents the random error caused by some uncertainty such as the effect of noise or unavoidable artificial factors.

However, when we deal with the FEM updating of structures, the estimated response variable $\hat{Ζ}\left(\Gamma \right)$ shown in (34) is obtained by the deterministic FEM analysis. Therefore, there is no random error **ε**_*L*2_ at all, and only the modeling error **ε**_*L*1_ exists (see (35)) which would be zero if the mathematical form of RSM could take infinite order polynomial or this mathematical form is exactly the same as the true model:

(35) $$\begin{array}{c} {\hat{Ζ}}_{j}\left(\Gamma \right)=\Theta b+\varepsilon_{L1}. \end{array}$$

As shown in the above equation, in theory, the estimated response variable $\hat{Ζ}\left(\Gamma \right)$ is equal to the true value if the modeling error **ε**_*L*1_ goes to zero, and the estimated response variable $\hat{Ζ}\left(\Gamma \right)$ has the unique value for each feature sample. Therefore, the response variable $\hat{Ζ}\left(\Gamma \right)$ is not the random variable. Following this logic, if the RSM is applied to replace the analytical FEM during model updating, all the methods introduced in Sections 4.3 and 4.4 cannot be worked any more since the generated RSM in this situation has no statistical meaning.

The above issue is easy to be ignored when the RSM is applied to update the FEM of structures. Since all the methods based on statistical theory do not work at all, the only way to assess the accuracy of the predictor of RSM under the given polynomial form is to check the difference between the estimated response variable and the true value. Meanwhile, the methods obtaining the coefficients of RSM are not the regression technique but the optimization methods such as least square method.

**Figure 2:**
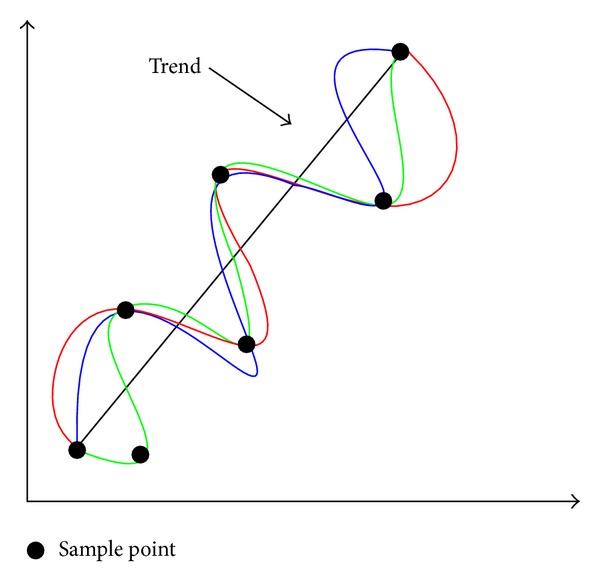
Sketch map of the idea of the Kriging model.

Different from the RSM, the Kriging model does not lack the random error. As shown in (18), the key difference between RSM and Kriging model lies in the random item *γ*_*j*_(***θ***_*v*_). The Kriging model consists of two parts, that is, trend term **f**_*j*_(***θ***_*k*_) and random term *γ*_*j*_(***θ***_*v*_). Based on the trend term shown in Figure 2, the random term is in charge of the accuracy of the Kriging predictor. The item *γ*_*j*_(***θ***_*v*_) is arbitrary function satisfying the equations from (20) to (22), which means that *γ*_*j*_(***θ***_*v*_) has no deterministic mathematical form. *γ*_*j*_(***θ***_*v*_) is usually a multivariable, so it is a stochastic process. Therefore, the approaches based on statistical theory work well for assessing and determining the mathematical form of Kriging model.

#### 4.5.2. Selection of the Metamodel for FEM Updating

In theory, the selection of the metamodel depends on the relationship between structural characteristics and structural parameters. During the procedure of FEM updating of structures, the structural parameters are the updating parameters that represent the modeling error between the analytical FEM and real structure. Using the Taylor series, the structural characteristics are described as the polynomial with respect to updating parameter, that is,

(36) $$\begin{array}{c} Ζ\left(\theta \right)\approx Ζ\left(\theta_{0}\right)+G\left(\theta_{0}\right)\left(\theta -\theta_{0}\right)+o\left(\Delta \theta^{2}\right), \end{array}$$

where ***θ***_0_ is the initial updating parameters and **G** represents the derivative matrix that is defined as follows:

(37) $$\begin{array}{c} G\left(\theta_{0}\right)=\left[\frac{\partial z_{j}}{\partial \theta_{k}}\right], \end{array}$$

where *j* = 1,2,…, *p* represents the number of the measured structural characteristics such as modal parameters and *k* = 1,2,…, *q* is the number of updating parameters. For the dynamic characteristics of structures, the derivative of the eigenvalue with respect to parameter *θ* is defined as [22]

(38) $$\begin{array}{c} \frac{\partial \lambda_{j}}{\partial \theta_{k}}=\varphi_{j}^{T}\left(-\lambda_{j}\frac{\partial M}{\partial \theta_{k}}+\frac{\partial K}{\partial \theta_{k}}\right)\varphi_{j}, \end{array}$$

where *λ*_*j*_ is the *j*th eigenvalue of structures.

As shown in the above equation, the nonlinear degree of the functional relationship shown in (36) changes with the variation of the updating parameters of structures. If the updating parameter is the coefficient of the element stiffness matrix, the second-order derivative shown in (36) goes to zero. In this case, the relationship between the response function and the updating parameter is approximate to linear function. If the length of the element *l* is taken as the updating parameter, the response function shown in (36) is the cubic function with respect to *l*. Furthermore, as we know, the larger the amounts of updating parameters increase, the higher the nonlinear degree of (36) becomes.

On the other hand, the nonlinear degree of the relationship shown in (36) also depends on the characteristics of structures. For example, there is the following relationship between the eigenvalue of structures and vibration frequency of structures:

(39) $$\begin{array}{c} \lambda_{j}\left(\theta \right)=\omega_{j}^{2}\left(\theta \right), \end{array}$$

where *ω*_*j*_ is the *j*th mode of structural frequency. According to above equation, comparing with *λ*_*j*_, the nonlinear degree between *ω*_*j*_ and ***θ*** is more serious.

Therefore, to select a reasonable metamodel for the purpose of FEM updating, it is significant to consider the nonlinear degree of the relationship between the characteristics of structures and the updating parameters. The nonlinear degree of the above relationship depends on the selected updating parameters as well as the structural characteristics. A reasonable metamodel should be determined case by case, and the complication of the structures, the selected updating parameters, and the goal of FEM updating (different characteristics of structures) are all the key factors for selecting the metamodel.

As discussed in the above two sections, comparing with the RSM and ANN, the reasons that we select the Kriging model for FEM updating are summarized as follows.

- A random item exists in the mathematical form of Kriging model, so all the methods based on statistical theory could be applied to assess and determine the Kriging predictor even though there is no random error for the deterministic FEM analysis.
- Kriging predictor is apt to handle the highly nonlinear function with respect to the updating parameters of FEM. The highly nonlinear relationship is always true for the practical complex structures, especially for the complicate or large-scale bridges, since the structural dynamic response is always the nonlinear function with respect to physical parameters of structures. The above two reasons make the Kriging model more proper to FEM updating than RSM.
- ANN is apt to build a metamodel with a large number of structural parameters, and the generation of ANN always needs a large number of training samples. However, when we build the optimization problem of FEM updating, the number of updating parameters has to be controlled into small-scale or medium-scale for the sake of avoiding the under-determined issue as far as possible. Therefore, ANN is rarely used to be metamodel for FEM updating.

### 4.6. Procedure of the Proposed Method

The procedure of the proposed method is described as follows.

- Build the reduced FEM by using the CMS method described in Section 3.
- Determine the set of feature samples Γ by using the methods introduced in Section 4.1.
- Obtain the set of characteristics of structures with respect to the selected feature samples **Ζ**(Γ) by using the condensed FEM obtained in step (i).
- Generate the Kriging model by using the Γ and **Ζ**(Γ).
- Determine the final Kriging model and assess the confidence level of the generated Kriging model by using the methods shown in Sections 4.3 and 4.4.
- Replace the analytical FEM with the generated Kriging model and update the FEM of structures by using the methods described in Section 2.

## 5. Experimental Example

In this section, we do not select a super huge structure as an example, so the regular method could be applied to this example. All the results of FEM updating of this example are obtained by the proposed method and the regular method in order to compare the performance of these two methods.

### 5.1. Description of an Arch Bridge Model

**Figure 3:**
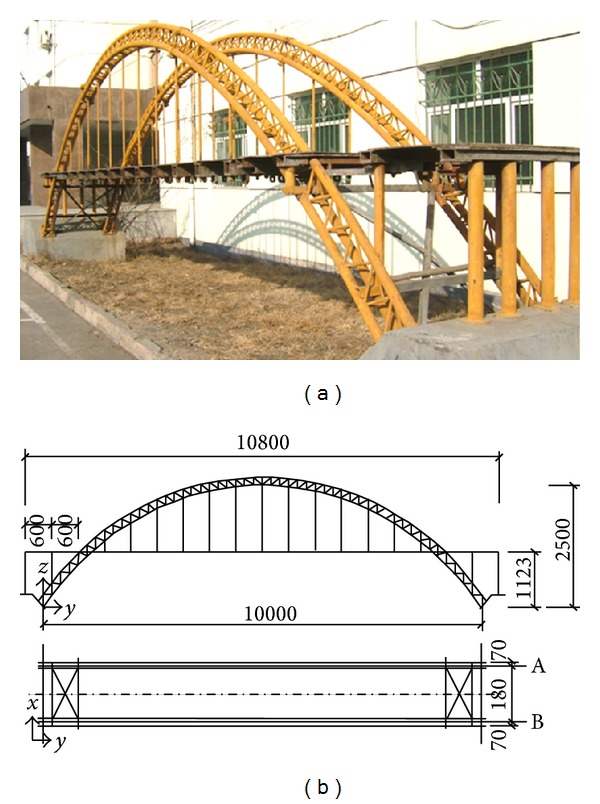
A 1 : 10 scaled arch bridge model (unit : mm).

As shown in Figure 3, this structure is a 1 : 10 scaled half-through arch bridge model, whose effective span and width are 10 m and 1.25 m, respectively. Each arch rib of this bridge consists of three concrete-filled steel tubes whose specifications are all Φ60 × 1.2 mm, and the material of each concrete-filled steel tube is composed of 16Mn steel and C50 concrete, respectively. Each arch circle consists of 13 suspenders, and each suspender is made up of 10 high strength steel wires whose specifications are all Φ5. The bridge deck is steel plate with 10 mm in thickness.

### 5.2. Dynamic Analysis of the Condensed FEM

**Figure 4:**
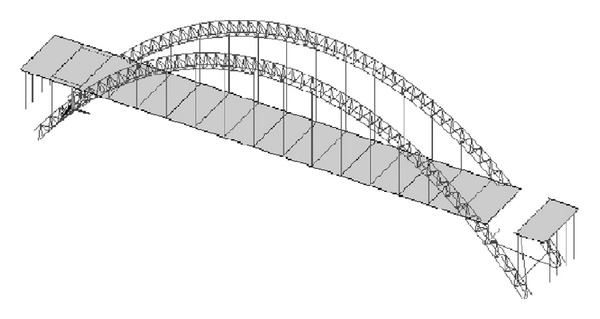
Un-condensed FE model of the bridge.

Firstly, the un-condensed FE model of this bridge structure was constructed with FE model program developed in MATLAB software environment. The beam element, pole element, and shell element were applied to model the arch ribs, suspenders and deck of this bridge model, respectively. Fixed support conditions were utilized to model the boundary conditions. At last there are total 378 nodes and 2172 DOF in the un-condensed FEM. The FEM of this bridge is shown in Figure 4.

**Figure 5:**
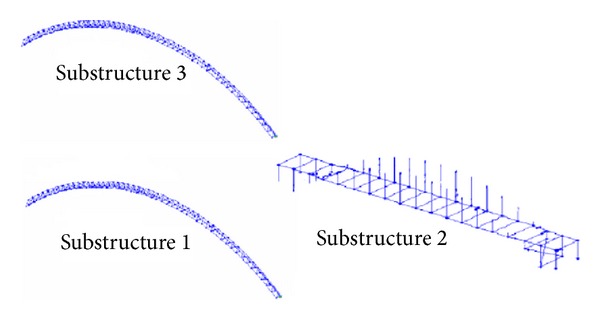
Three substructures.

Secondly, the condensed FEM of this structure was also built by dividing the whole bridge into three substructures as shown in Figure 5. With the improved CMS method, the condensed FE model of this bridge was built. Since the first 40 modes of all the components and interfaces were utilized to build the condensed model (i.e., the values of *n* in (13) and (14) are taken 40), the total DOF of condensed model is 200 that are less than the total DOF of original FEM. As shown in Table 1, the accuracy of the CMS method is good enough, and the biggest error between the original FEM and the condensed FEM is less than 0.5%.

### 5.3. Modal Test of an Arch Bridge Model

**Figure 6:**
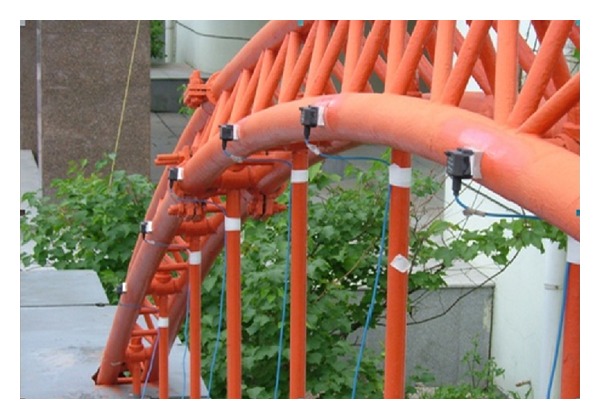
Placement of accelerate sensors.

**Figure 7:**
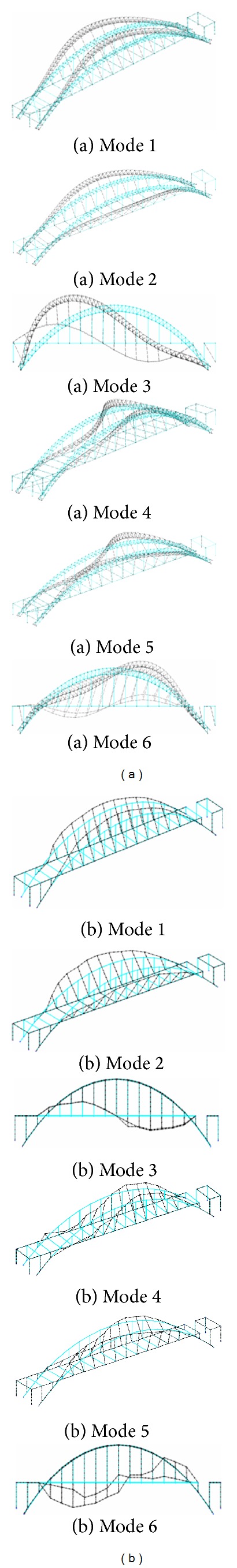
(a) Analytical modal shapes and (b) measured modal shapes.

Dynamic test on this structure was performed, and eigensystem realization algorithm (ERA) [23] was applied to identify the modal parameters by using the measured accelerations. During the modal test, accelerometers were placed on the bridge deck and arch ribs in order to measure the structural vibration, as shown in Figure 6. The comparison between the identified dynamic properties and analytical results is shown in Figure 7 and Table 2.

### 5.4. FEM Updating of an Arch Bridge Model

#### 5.4.1. Selection of Parameters to Be Updated

Theoretically, all structural parameters could be selected for updating parameters in model updating procedure. However, it is computationally costly if too many parameters are included. Therefore, the set of parameters to be updated should be selected with caution.

As shown in Table 2, the relatively big differences between the analytical and measured frequencies lie in mode 1, mode 2, and mode 5, and the modal shapes of these three modes are all the vibration of arch ribs. Therefore, the modeling error of arch ribs is believed to be the main factor causing the frequency error. Furthermore, for the simplification of modeling, the coupled effect between concrete and steel tube is not considered, which also caused some modeling error of arch ribs. Owing to the above reasons, the stiffness and mass of arch ribs, total of 4 parameters, are selected as the updating parameters, as listed in Table 3.

#### 5.4.2. Generation of the Kriging Model

According to the above section, the stiffness and mass of arch ribs were selected as the updating parameters. To realize this in the analytical FE model, we assigned a coefficient to the stiffness matrix or mass matrix of the substructure that was planned to be updated. These coefficients are the updating parameters (the initial value of each updating parameter takes one).

As shown in Table 2, there is no big difference between the measured modal shapes and the analytical modal shapes, so only the first 6 modes of frequencies are deemed as the goal of FE model updating.

Using the method described in Section 4.1, the set of feature samples Γ (80 samples) were generated, and the value of each stiffness sample belongs to [0.5, 1.5] and the one of mass sample goes to [0.9, 1.1]. Since the modeling error mainly lies in the stiffness of arch ribs, so we defined the ±50% change of the stiffness coefficient of each arch rib in order to build a big enough feasible region for the optimization of FEM updating. Considering the physical meaning, the mass coefficient of each arch rib took only ±10% variation. The corresponding set of response function **Ζ**(Γ) was obtained by using the condensed FEM obtained in Section 5.2. With the generated Γ and **Ζ**(Γ), the metamodel was built.

**Figure 8:**
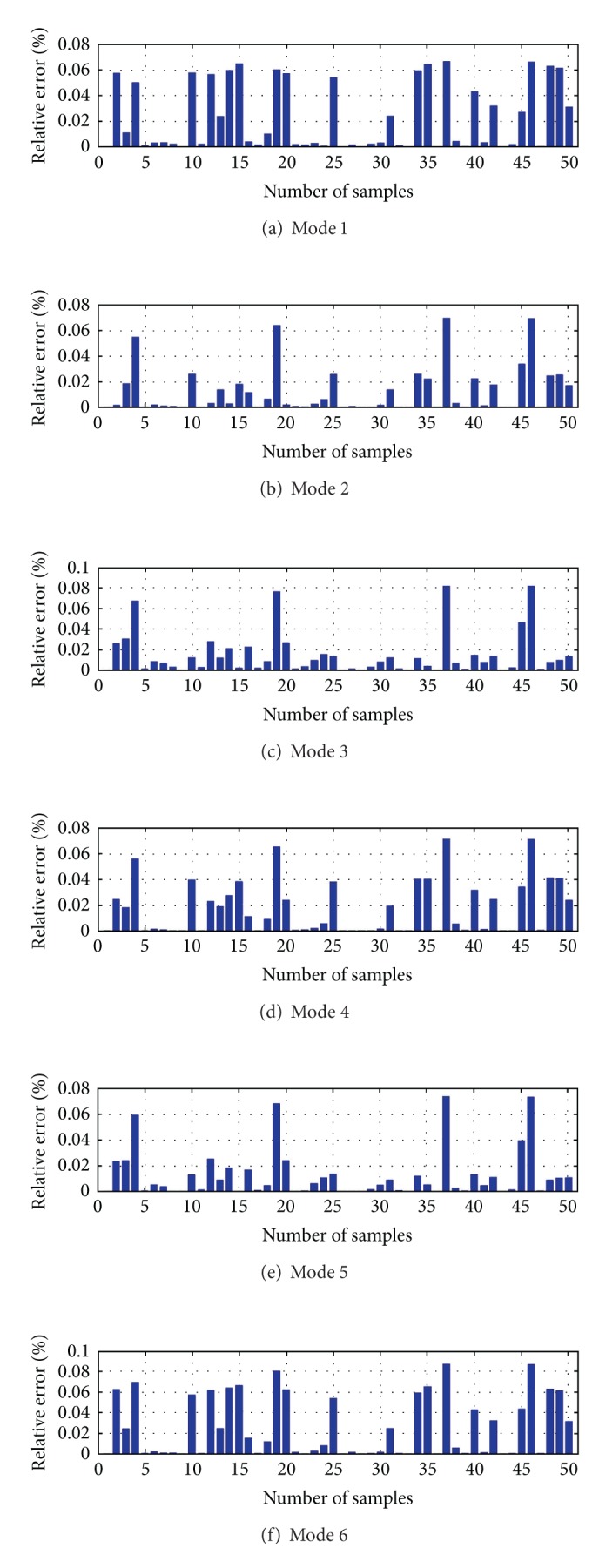
Frequency difference between Kriging model and condensed FEM.

**Figure 9:**
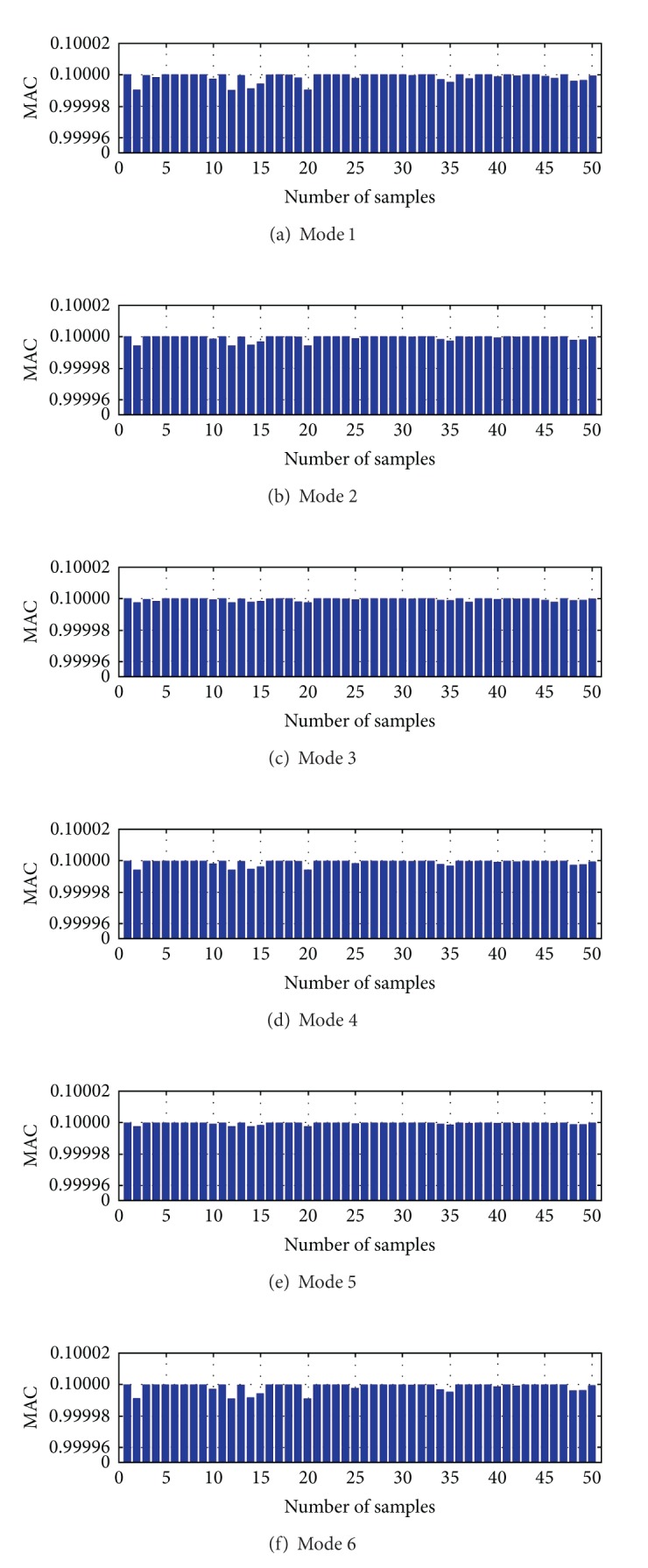
Modal shapes difference between Kriging model and condensed FEM (note: MAC represents the matching degree of modal shapes obtained by two different models).

During the generation of the Kriging model, the trend item in (18) took the constant and the random item took the Gauss function. Using the same Γ and **Ζ**(Γ), the model coefficients of Kriging model were obtained. The methods described in Sections 4.3 and 4.4 were used to assess the generated Kriging model. We used 50 extra samples to check the performance of the generated Kriging model. The comparison of the frequency and modal shapes between Kriging model and condensed analytical FEM is shown in Figures 8 and 9. Based on these results, it is conducted that the accuracy of the generated Kriging model is good enough, and this Kriging model is applied to update the FE model of the arch bridge model.

#### 5.4.3. Results of FE Model Updating

Using the method described in Section 2, both the metamodel obtained by the proposed two-level method and the regular method based on the un-condensed FEM are applied to update the FEM of this bridge. Updated parameters before and after model updating are listed in Table 3. The updated parameters obtained by two models are almost the same, so the proposed metamodel can keep the high accuracy with the original FEM. With the proposed method, the dynamic properties of this bridge before and after FEM updating are listed in Table 4. The results of Table 4 show that the frequency obtained by the updated FEM and the measured results matches up very well. The efficiency of the proposed method is much higher than the regular way as shown in Table 5.

Since this bridge model is a symmetrical structure, in theory, there should be some repeated roots in the analytical frequencies such as the pair between mode 1 and mode 2 and the pair between mode 4 and mode 5, as listed in Table 2. However, repeated roots cannot be found in the measured frequencies. Therefore, the stiffness difference between two arch ribs is the main reason for this phenomenon, and this is also consistent with the updated results.

## 6. Conclusions

To deal with the FEM updating of complex structures, a two-level method that combined the CMS method and Kriging model is proposed in this study. Herein, we consider the situation that it is difficult for the analytical FEM of large-scale structures to carry out the dynamic analysis directly, which means the generation of metamodel is also impossible since the dynamic analysis based on the analytical FEM has to be implemented repeatedly during the procedure of building. With the proposed two-level method, the above issue can be solved successfully, and the implementing of FEM updating of complex structures is also ensured. The Kriging model is deemed as the metamodel for FEM updating in this study. The reasons for this selection are explained by comparing the performance of Kriging model and RSM. Meanwhile, some key issues of the application of metamodel to FEM updating are also discussed. Finally, it is demonstrated that the proposed method is promising for the FEM of complex structures by updating the FEM of an arch bridge model with the measured modal parameters.

## Figures and Tables

**Table 1:** Comparison of dynamic properties between condensed and uncondensed FEM.

Mode	Frequency (Hz)		Frequency error (%)	MAC*
	Condensed FE model	Uncondensed FE model		
1	6.91	6.88	0.44	0.91
2	7.13	7.12	0.14	0.90
3	11.00	10.96	0.37	0.92
4	16.93	16.91	0.12	0.90
5	17.20	17.19	0.06	0.92
6	20.54	20.60	0.29	0.93

*MAC represents the modal assurance criterion.

**Table 2:** Comparison of modal parameters between ERA method and the condensed FEM.

Mode	Frequency (Hz)		Frequency error (%)	MAC
	Condensed FE model	ERA method		
1	6.91	6.47	6.34	0.94
2	7.13	8.00	11.00	0.94
3	11.00	10.54	3.98	0.96
4	16.93	16.67	1.44	0.80
5	17.20	19.37	11.25	0.89
6	20.54	21.64	5.05	0.87

**Table 3:** Updating parameters before and after model updating.

Updating parameters	Before updating	After updating	
		Proposed meta-model	Original FEM
Stiffness coefficient of substructure 1	1.00	1.15	1.17
Mass coefficient of substructure 1	1.00	0.93	0.95
Stiffness coefficient of substructure 3	1.00	1.07	1.08
Mass coefficient of substructure 3	1.00	0.96	0.96

**Table 4:** Comparison of the results before and after updating (proposed method).

Mode	Identified frequencies	Before updating		After updating	
		Frequency (Hz)	Error (%)	Frequency (Hz)	Error (%)
1	6.47	6.91	6.34	6.74	4.49
2	8.00	7.13	11.00	7.71	3.73
3	10.54	11.00	3.98	11.11	3.98
4	16.67	16.93	1.44	16.91	1.44
5	19.37	17.20	11.25	18.42	4.79
6	21.64	20.54	5.05	22.63	4.80

**Table 5:** Comparison of efficiency between the proposed method and the regular method.

Calculating time (unit: minute)			
Dynamic analysis		FE model updating	
Condensed FEM	Uncondensed FEM	Proposed method	Regular method
0.79	7.67	121.30	1265.55

## Appendix

According to the content of Section 4, we assume the real *Z*_*j*_(***θ***_*v*_) is defined as

(A.1) $$\begin{array}{c} Z_{j}\left(\theta_{v}\right)=f_{j}{\left(\theta_{v}\right)}^{T}\beta_{j}+a\left(\rho_{j},w,\theta_{v}\right), \end{array}$$

where *a*(**ρ**_*j*_, **w**, ***θ***_*v*_) is the random error term.

Furthermore, the estimation of **Ζ**_*j*_(Γ) is supposed as

(A.2) $$\begin{array}{c} {Ζ}_{j}\left(\Gamma \right)=AZ=A\left(FB+\Sigma \right), \end{array}$$

where **A** = [**α**_1_,…,**α**_*N*_]^*T*^, Σ = [**γ**(***θ***_1_),…,*γ*(***θ***_*N*_)]^*T*^ **B** = [**β**_1_, **β**_2_,…], and **F** = [**f**(***θ***_1_),…,**f**(***θ***_*N*_)]^*T*^.

Using above equation, the ${\hat{Z}}_{j}\left(\theta_{v}\right)$ shows in (18) is rewritten by

(A.3) $$\begin{array}{c} {\hat{Z}}_{j}\left(\theta_{v}\right)=\alpha_{j}^{T}\cdot \left[F\cdot \beta_{j}+\gamma_{j}\left(\theta_{v}\right)\right]. \end{array}$$

Therefore, the error between ${\hat{Z}}_{j}\left(\theta_{v}\right)$ and *Z*_*j*_(***θ***_*v*_) is defined as

(A.4) ε=Z^j(θv)−Zj(θv)=αjT[Fβj+γ(θv)]−(fj(θv)Tβj+γj(θv))=(αjTF−fj(θv)T)βj+αjTγ(θv)−γj(θv).

To keep the unbiased estimator, the following equation is obtained:

(A.5) $$\begin{array}{c} F^{T}\alpha_{j}-f_{j}\left(\theta_{v}\right)=0. \end{array}$$

And then the error defined in $\left (\text {A}.4\right )$ is simplified as

(A.6) $$\begin{array}{c} \varepsilon =\alpha_{j}^{T}\gamma \left(\theta_{v}\right)-\gamma_{j}\left(\theta_{v}\right). \end{array}$$

So the mean square error of the estimation is defined as

(A.7) Vj(θv)=E[(αjTγ(θv)−γj(θv))2]=E(αjTγ(θv)·γ(θv)Tαj  −2αjTγ(θv)γj(θv)+γj2(θv)).

With the definition shown in (20), the above equation is rewritten as

(A.8) Vj(θv)=Var⁡(γj(θv))·(1+αjTCor(γ(θv),γ(θv))αj       −2αjTCor(γ(θv),γj(θv))).

Minimizing the *V*_*j*_(***θ***_*v*_) with the constraints of the unbiased estimation, the following optimal objective function is defined as

(A.9) J(α,η)=Var⁡(γj(θv))·(1+αjTCor(γ(θv),γ(θv))αj       −2αjTCor(γ(θv),γj(θv)))−ηjT·(FTαj−fj(θv)),

where **η** is the Lagrangian function, and the following results are obtained by ∂*J*/∂**α** = 0:

(A.10) ηj¯=(FTCor(γ(θv),γ(θv))−1F)−1·(f(θv)−FTCor(γ(θv),γ(θv))−1γ),αj=Cor(γ(θv),γ(θv))−1(γ+Fηj¯),

where $\bar{\eta_{j}}=\left(1/2\mathrm{Var}\left(\gamma_{j}\left(\theta_{v}\right)\right)\right)\eta_{j}$. Then, ${\hat{Z}}_{j}\left(\theta_{v}\right)$ is obtained as

(A.11) Vj(θv)=E[(αjTγ(θv)−γj(θv))2]=E(αjTγ(θv)·γ(θv)Tαj  −2αjTγ(θv)γj(θv)+γj2(θv)),

where

(A.12) β∗=(FTCor(γ(θv),γ(θv))−1F)−1·FTCor(γ(θv),γ(θv))−1Z,η∗=Cor(γ(θv),γ(θv))−1(Z−F·β∗).

To obtain the coefficients **ρ**_*j*_ of the correlation function, the following maximum likelihood function is defined as

(A.13) L´(ρj ∣ Z) =−(Z−F·β∗)TCor(γ(θv),γ(θv))−1(Z−F·β∗)2Var⁡(γ(θv)).

With $\left (\text {A}.13\right )$, the following results are obtained by solving $\partial \left(\overset{´}{\text{L}}\left(\rho_{j}|Z\right)\right)/\partial \left(\theta_{v}\right)=0$:

(A.14) β∗=(FTCor(γ(θv),γ(θv))−1F)−1·FTCor(γ(θv),γ(θv))−1Z,Var⁡(γ(θv))=(Z−F·β∗)T(Z−F·β∗)N.

Since both **β*** and Var⁡(**γ**(***θ***_*v*_)) are the function of **ρ**_*j*_, the following optimal objective function is defined as

(A.15) $$\begin{array}{c} \max\left\{-\frac{N}{2}\ln\left(\mathrm{Var}\left(\gamma \left(\theta_{v}\right)\right)\right)-\frac{1}{2}\ln\left(\left|\text{Cor}\left(\gamma \left(\theta_{v}\right),\gamma \left(\theta_{v}\right)\right)\right|\right)\right\}. \end{array}$$

Finally, the **ρ**_*j*_ is obtained by solving the above optimization problem, and then the Kriging model is determined.
